# Serum-free microcarrier based production of replication deficient Influenza vaccine candidate virus lacking NS1 using Vero cells

**DOI:** 10.1186/1472-6750-11-81

**Published:** 2011-08-11

**Authors:** Allen Chen, Swan Li Poh, Christian Dietzsch, Elisabeth Roethl, Mylene L Yan, Say Kong Ng

**Affiliations:** 1 Bioprocessing Technology Institute, Agency for Science, Technology and Research (A*STAR), 20 Biopolis Way, #06-01, Centros, Singapore 138668, Singapore; 2 Avir Green Hills Biotechnology, Forsthausgasse 11, 1200 Vienna, Austria

**Keywords:** Influenza, Vero, Microcarrier, NS1, Bioreactor

## Abstract

**Background:**

Influenza virus is a major health concern that has huge impacts on the human society, and vaccination remains as one of the most effective ways to mitigate this disease. Comparing the two types of commercially available Influenza vaccine, the live attenuated virus vaccine is more cross-reactive and easier to administer than the traditional inactivated vaccines. One promising live attenuated Influenza vaccine that has completed Phase I clinical trial is deltaFLU, a deletion mutant lacking the viral Nonstructural Protein 1 (NS1) gene. As a consequence of this gene deletion, this mutant virus can only propagate effectively in cells with a deficient interferon-mediated antiviral response. To demonstrate the manufacturability of this vaccine candidate, a batch bioreactor production process using adherent Vero cells on microcarriers in commercially available animal-component free, serum-free media is described.

**Results:**

Five commercially available animal-component free, serum-free media (SFM) were evaluated for growth of Vero cells in agitated Cytodex 1 spinner flask microcarrier cultures. EX-CELL Vero SFM achieved the highest cell concentration of 2.6 × 10^6 cells/ml, whereas other SFM achieved about 1.2 × 10^6 cells/ml. Time points for infection between the late exponential and stationary phases of cell growth had no significant effect in the final virus titres. A virus yield of 7.6 Log_10 _TCID_50_/ml was achieved using trypsin concentration of 10 μg/ml and MOI of 0.001. The Influenza vaccine production process was scaled up to a 3 liter controlled stirred tank bioreactor to achieve a cell density of 2.7 × 10^6 cells/ml and virus titre of 8.3 Log_10 _TCID_50_/ml. Finally, the bioreactor system was tested for the production of the corresponding wild type H1N1 Influenza virus, which is conventionally used in the production of inactivated vaccine. High virus titres of up to 10 Log_10 _TCID_50_/ml were achieved.

**Conclusions:**

We describe for the first time the production of Influenza viruses using Vero cells in commercially available animal-component free, serum-free medium. This work can be used as a basis for efficient production of attenuated as well as wild type Influenza virus for research and vaccine production.

## Background

Influenza virus is a major health concern that has huge impacts on the human society. Historically responsible for millions of deaths in pandemics, the virus also causes seasonal outbreaks during colder months in temperate regions which annually result in up to 500,000 deaths worldwide [1]. Although antiviral drugs for acute treatment are available in some countries, vaccination remains as one of the most effective ways to mitigate this disease.

Both inactivated vaccine and the live attenuated Influenza vaccines are commercially available. Although the live attenuated virus vaccine has been used in Russia since the 1960s [2], concerns regarding safety and possible virus shedding have precluded it from use in the rest of the world until recently: In 2003, a cold adapted, egg grown, live attenuated influenza virus vaccine by MedImmune was licensed for use in the US [3,4]. Live attenuated virus vaccines have the added advantage of being more cross-reactive than traditional inactivated vaccines [5-7]. This type of vaccine is also easier to administer, since it is delivered in the form of nasal sprays, compared to injections for the traditional inactivated influenza vaccines.

One promising live attenuated Influenza that has completed Phase I clinical trial is deltaFLU, a deletion mutant lacking the viral Nonstructural Protein 1 (NS1) gene developed by Avir Green Hills Biotechnology [8-12]. As NS1 is an interferon antagonist [13], the NS1 deletion virus is replication defective in interferon competent host systems, enabling its use as a live attenuated vaccine [9-11]. Another consequence of this gene deletion is that this virus vaccine can only propagate effectively in cells with a deficiency in the interferon-mediated antiviral response [8]. Vero (African Green Monkey kidney) is one such cell line as the gene locus encoding the main Type I interferons, Interferon α and β, are missing from its genomic DNA [14,15]. Consequently, it has been previously demonstrated that the NS1 deletion Influenza virus grows efficiently in Vero cells, but not in MDCK or mice [8,16,17]. This NS1 deletion virus is also interesting because it may find applications in cancer therapy [18,19] and other prophylactics [20].

Regardless of vaccine type (inactivated or live attenuated), virus vaccine production requires the initial step of propagating the Influenza viruses carrying the haemaglutinin and neuraminidase antigens of the strains that the vaccine is providing prophylaxis for. These viruses are traditionally propagated in embryonated hen eggs. Two important limitations of this process are the inflexible supply of high quality specific pathogen free (SPF) eggs and possible low titres of emerging viruses, such as the highly pathogenic Influenza A (H5N1) strain. To provide an alternative to egg-based vaccine production, mammalian cell culture based production has been developed in recent years [21]. This provides a flexible and scalable platform that can make use of existing biopharmaceutical infrastructure for Influenza vaccine production.

Three cell lines commonly used for Influenza virus production are the PER.C6 cells, MDCK (Madin-Darby Canine Kidney) and Vero (African Green Monkey Kidney). All three cell lines can be grown in serum-free media. While PER.C6 and MDCK can be cultured in suspension [22,23], microcarriers are commonly used for culturing MDCK [22,24-30] and Vero cells [31-34] because these cell lines are typically anchorage dependent. The seasonal and pandemic Influenza vaccine produced in MDCK cells by Novartis has gained various regulatory approvals in 2007 and 2009 respectively, while those produced in Vero cells by Baxter has also gained approvals in 2010 and 2009 respectively.

Although bioreactor production of Influenza virus has been developed, serum-free production processes described in literature commonly use proprietary in-house cell culture media [24,25,29,30,32]. To our knowledge, there are a few reports describing Influenza virus production using MDCK cells in commercially available serum-free medium [21,26-28], while that using Vero cells is described in only one recent report [35] although the medium used contains animal components. Related literature described serum-free media for Vero cells [36,37] and microcarrier bioreactor processes for the production of other viruses using Vero cells [37-46]. It is important to bridge this gap to provide a scalable animal-component free, serum-free platform for researchers and academics to produce different Influenza viruses using Vero cells.

In this report, we describe for the first time, a scalable bioreactor process for the production of Influenza A virus lacking NS1 in Vero cells using commercially available animal-component free, serum-free media. We chose to use Cytodex 1 microcarriers for our bioreactor cell culture, since this microcarrier has been previously reported for Vero cells [31,33,34,39-43,45,46]. We evaluated five commercially available animal-component free, serum-free media for Vero cells by comparing the cell yield in these media. The medium giving the highest cell densities was then used to develop the bioreactor process for Influenza virus production. This involved studies of parameters that will affect the virus production process, namely trypsin concentration, time-point of infection (TOI), and multiplicity of infection (MOI). These parameters were validated in classical stirred tank bioreactor processes. Finally, we also compared the production of the NS1 truncated Influenza A virus with that of the corresponding wild type Influenza A virus.

## Results and Discussion

### Growth kinetics of Vero cell microcarrier culture in different SFMs

**Figure 1:**
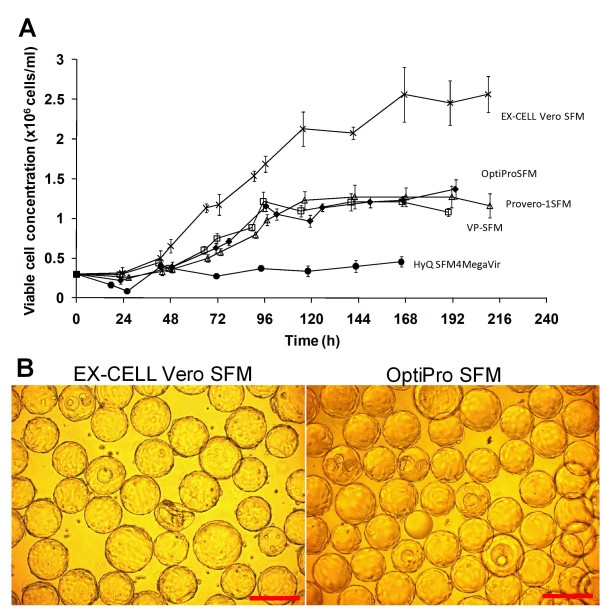
**Vero cell microcarrier cultivation in different commercial serum-free media**. (A) Comparison of Vero cell growth on Cytodex 1 microcarriers in five different serum-free media, OptiPro SFM, EX-CELL Vero SFM, VP-SFM, Provero-1 and HyQ SFM4MegaVir. The experiment was performed in duplicate using 250 ml spinner flasks with 3 g/l Cytodex 1 microcarriers. The viable cell concentrations of one representative run are shown here. (B) Phase contrast images of cells cultured on Cytodex 1 microcarriers in EX-CELL Vero SFM and OptiPro SFM at day 8. Scale bars indicate 200 μm.

The growth kinetics of Vero cells in the 5 commercially available animal-component free, serum-free media (SFM) were evaluated in 250 ml spinner flasks. The media evaluated were OptiPro SFM (Invitrogen), VP-SFM (Invitrogen), EX-CELL Vero SFM (SAFC Bioscience), Provero-1 (Lonza) and HyQ SFM4MegaVir (HyClone). The results are presented in Figure 1A. Poor attachment of cells to microcarriers and poor cell growth was observed in HyQ SFM4MegaVir, which consequentially yielded a low cell concentration of 4.5 × 10^5 ^cells/ml. OptiPro SFM, VP-SFM and Provero-1 SFM displayed similar cell growth profiles, yielding cell concentrations of 1.2 × 10^6 ^cells/ml with viability above 90% on days 4 or 5. Growth of Vero cells in EX-CELL Vero SFM was the highest achieving 2.6 × 10^6 ^cells/ml (97% viability) on day 7.

**Figure 2:**
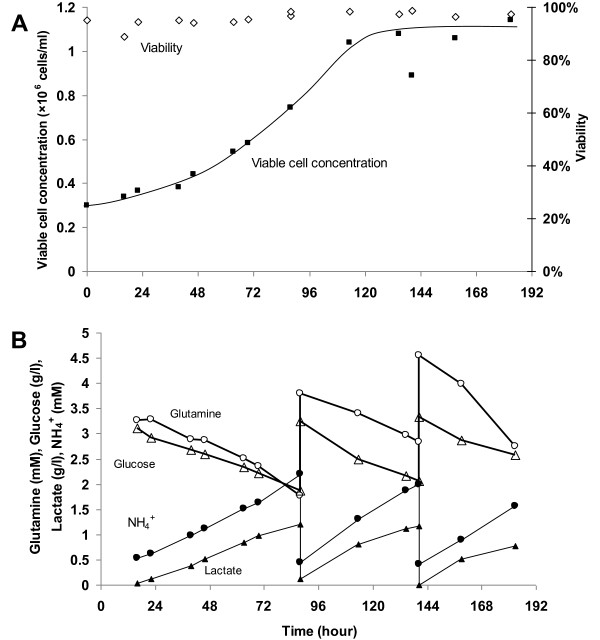
**Vero cell growth on microcarriers in OptiPro SFM with two medium exchanges**. The experiment was performed in duplicate using 250 ml spinner flasks with 3 g/l Cytodex 1 microcarriers. (A) Cell growth profile and (B) Metabolite profiles of glucose, lactate, glutamine and ammonium.

Previous studies have reported that serum-free medium enriched with serum were able to achieve such high cell concentrations in batch cultivation of Vero cells when compared to serum-free media [47-49]. The difference in the maximum cell concentration reached was explained by the higher cell death rate in the serum-free medium, possibly caused by the lack of protective effect of serum, depletion of essential nutrient and accumulation of toxic metabolites [50]. To investigate whether this phenomenon can be replicated by providing nutrients and removing toxic metabolites for cells cultivated in OptiPro SFM, cell cultivation with medium exchanges was carried out (Figure 2). However, the higher cell density observed in EX-CELL Vero SFM was not attainable with this strategy. Comparing the cell morphology in these two media, a more compact cell monolayer was observed in EX-CELL Vero SFM when compared to cells cultured in OptiPro SFM (Figure 1B). Hence, the higher cell concentration in EX-CELL Vero SFM was not due to multilayer of cells, but a difference in cell morphology.

Comparing the maximum cell densities observed in this report with other studies involving Vero cell cultivation on Cytodex 1 using serum-free media, Souza *et al*. [41] obtained comparable maximum cell density of 1.6 × 10^6 ^cells/ml in VP-SFM, while Rourou *et al*. and Tiwari *et al*. [39,40] reached 2.6 × 10^6 ^cells/ml and 2.1 × 10^6 ^cells/ml respectively in the same media. The reason for these higher maximum cell densities was not clear in these reports. However, since the same Cytodex 1 concentration of 3.0 g/l was used, we speculate that these may also be due to a more compact cell monolayer, similar to our observation in EX-CELL Vero SFM cultures. The difference in cell morphology may be greater in the former study [42] because an even higher maximum cell density of 5 × 10^6 ^cells/ml on 3.0 g/l Cytodex 1 was reported in perfusion mode. In contrast, Silva *et al*. [42] reported a maximum cell density of 1 × 10^6 ^cells/ml for Vero cells cultivated in EX-CELL Vero SFM using the same microcarrier concentration. This may be due to a lack of adaptation from serum containing medium since the cells were directly seeded into EX-CELL Vero SFM for infection 24 h later. Other reports of Vero cultivation in different SFM typically achieved less than 2 × 10^6 ^cells/ml [35,43,44,46], except one using a proprietary medium [45].

As higher maximum cell densities were observed in other studies using VP-SFM [39,40], one possible explanation for the observed change in cell morphology may be differences in cell handling during adaptation to SFM. As such, relevant characteristics such as tumorigenicity of the cells should be investigated before these cells are used to produce clinical materials. Another perspective to investigate this phenomenon is to look at the available information on the components and formulation of these media: We observed higher starting glucose and amino acid contents in EX-CELL Vero SFM and VP-SFM compared to OptiPro SFM, as well as undefined plant hydrolysates and recombinant proteins. We speculate that these differences may also play an integral role in enabling the higher cell concentrations in EX-CELL Vero SFM and VP-SFM cultures.

**Figure 3:**
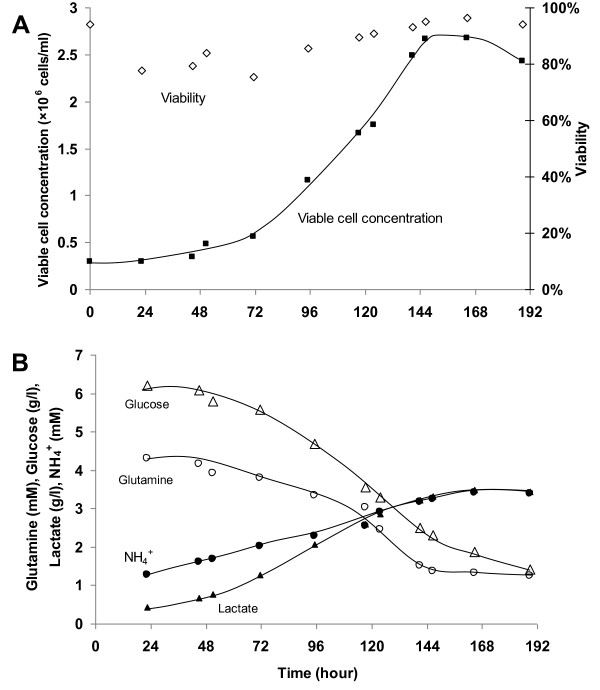
**Cultivation of Vero cells on microcarriers in a 3 L stirred tank bioreactor using EX-CELL Vero SFM with 3 g/l Cytodex 1**. The experiment was performed in triplicate. (A) Cell growth profile and (B) Metabolite profiles of glucose, lactate, glutamine and ammonium.

Since viable cell yield in EX-CELL Vero SFM was highest, it was chosen for our subsequent studies. Vero cell cultivation in EX-CELL Vero SFM was scaled up in a 3 L stirred tank bioreactor for validation. The results are presented in Figure 3. Despite the longer lag phase, cell yield of 2.7 × 10^6 ^cells/ml (93% viability) were comparable to those achieved in spinner flask (Figure 1). 3.9 g/l of glucose and 2.9 mM of glutamine were consumed and 2.9 g/l lactate and 2.2 mM ammonium were produced by day 6 (Figure 3B) when peak cell density was reached. The maximal specific growth rate was calculated to be 0.019 h^-1^, which is similar to that of our spinner flask culture (0.017 h^-1^) and those from previous studies with other SFM (0.026 h^-1 ^[48,50], 0.023-0.033 h^-1 ^[39]).

### Parameters for Influenza infection: trypsin concentration, multiplicity of infection (MOI) and time-point of infection (TOI)

**Figure 4:**
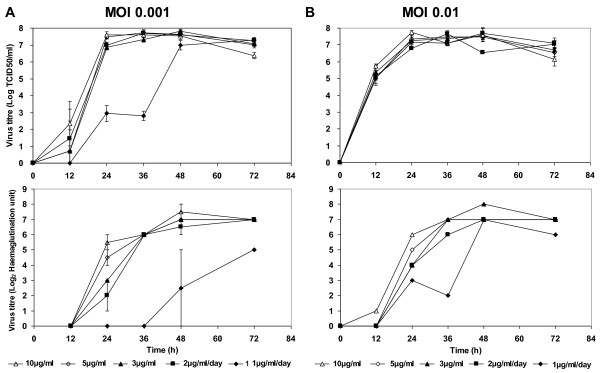
**Establishing trypsin concentration and MOI for ΔNS1 H1N1 Influenza virus production in Vero cells cultured on microcarriers with EX-CELL Vero SFM**. Vero cells were first cultivated using 250 ml spinner flasks with 3 g/l Cytodex 1 microcarriers. Upon confluency, 80% of culture medium was replaced with fresh medium. 5 ml of this culture was transferred to each well on suspension 6-well plates to test the different trypsin concentrations and MOI for infection. Trypsin concentrations used were 10 μg/ml, 5 μg/ml, 3 μg/ml, 2 μg/ml/day and 1 μg/ml/day. Virus titres obtained using TCID_50 _(Top) and haemagglutination assays (Bottom) at (A) MOI of 0.001 and (B) MOI of 0.01. Virus titres shown represent mean values obtained from two replicate wells. Error bars indicate the standard deviation of the experiment.

Trypsin is essential for the replication of some Influenza virus strains. To assess the effect of trypsin concentration on the amplification of ΔNS1 H1N1, we performed small scale infections of microcarrier cultures in 6-well suspension culture plates. Vero cells were first cultivated in 250 ml spinner flask in EX-CELL Vero SFM. When the culture reached 2 × 10^6 ^cells/ml, cells were transferred into 6-well suspension culture plates for infections. The wells were supplemented with different trypsin concentrations of 3 μg/ml, 5 μg/ml and 10 μg/ml in duplicates. To investigate the possibility of using lower trypsin concentrations by daily feeding, 2 sets of wells were supplemented with trypsin at 1 μg/ml/day and 2 μg/ml/day respectively. MOI of 0.01 and 0.001 were used in this experiment to concurrently assess the effect of this parameter on virus amplification. Samples were harvested 12 hourly and virus titres were determined by haemagglutination (HA) and TCID_50 _assays (Figure 4).

Virus production with 1 μg/ml/day of trypsin yielded lower HA titres for both MOI tested, although peak TCID_50 _titres were similar albeit at a later time-point for MOI of 0.001. This suggests that ΔNS1 H1N1 virus production was limited by trypsin at a concentration of 1 μg/ml. On the other hand, virus production using 3 μg/ml, 5 μg/ml and 10 μg/ml trypsin, as well as daily trypsin feed at 2 μg/ml/day, yielded high peak virus titres between 7.5 and 8.0 Log_10 _TCID_50_/ml for both MOI tested. Since the assay has a standard deviation of 0.4 Log_10 _TCID_50_/ml, the peak virus titres with the above conditions were not significantly different. However, infection using 10 μg/ml trypsin resulted in higher virus titres at the 24 h time point for both MOI, implying a faster virus amplification process. Similar observations were also described in literature [27,35]. As live (TCID_50_) virus titres were reported to decrease with time [27,35], a faster virus amplification process with 10 μg/ml trypsin is beneficial for the production of live attenuated virus vaccines such as ΔNS1 H1N1. Hence 10 μg/ml trypsin and MOI of 0.001 (for lower amounts of virus inoculums during vaccine production) were used in the subsequent experiments.

**Figure 5:**
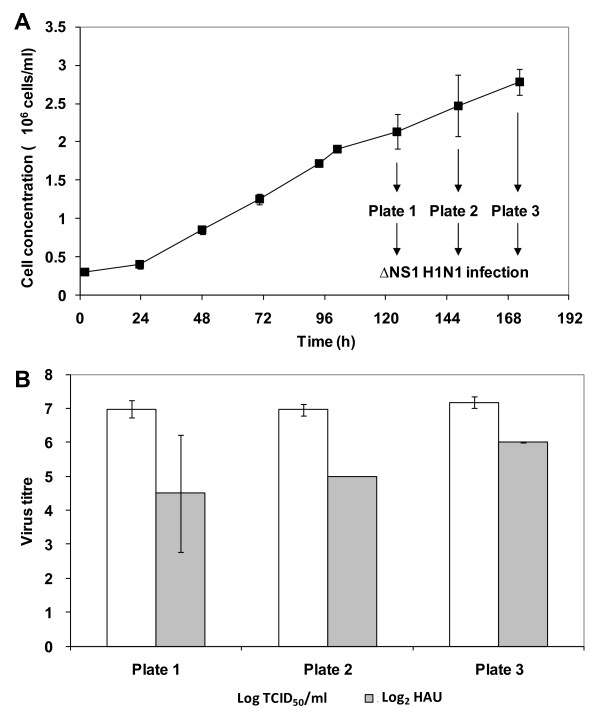
**Establishing time-point of infection (TOI) for ΔNS1 H1N1 Influenza virus production in Vero cells cultured on microcarriers with EX-CELL Vero SFM**. (A) Growth curve of Vero cells cultivated using 250 ml spinner flasks with 3 g/l Cytodex 1 microcarriers. Cells were infected at day 5, day 6 and day 7 as depicted, by transferring 5 ml of the culture to each well on suspension 6-well plates. (B) Virus titre was measured at 48 hours post-infection for all TOI. Each bar represents the mean of the virus titres yielded from two runs using MOI of 0.001. The error bars indicate the standard deviation of the two runs.

For cell-based Influenza virus production, culture infection is typically performed at a time-point close to when the peak cell density is reached without time-point of infection (TOI) studies [26-28,32,35]. However, varying TOI has been shown to increase titres of other viruses [38,41]. In addition to a difference in cell densities at the different TOI, the state of the cells at the different phases of cell culture may also affect virus production. To determine the effect of TOI for ΔNS1 H1N1 infection, 3 time-points at the late exponential to stationary phase were tested. Vero cells were first cultivated on microcarriers in spinner flask using the same condition as previously described. Figure 5A shows the average cell concentration measured from two spinner flasks. At days 5, 6, and 7, cells were transferred to 6-well suspension culture plates and infected at MOI 0.001 with 10 μg/ml of trypsin. The peak virus titres, obtained 48 h post-infection in all the 6-well plates, are shown in Figure 5B.

The peak virus titres from the day 7 samples were slightly higher than those from other time-points. Day 7 is also when the peak cell density of 2.8 × 10^6 ^cells/ml was reached. However, statistical analysis shows that the virus titres obtained in these three time points are not significantly different (p-value > 0.5). This suggests that time-point of infection between the late exponential to stationary phases of cell growth, with the different cell densities from 2.1 to 2.8 × 10^6 ^cells/ml, does not significantly affect virus yield. The lack of increase in virus titres with infections at higher cell densities is also known as the "cell density effect". This was first observed study by Wood *et al*. [51] and later reported in other virus production system [39,52-55]. While nutrient limitation and unknown inhibitory factors generated during the process can account for most cases (adenovirus [52,53], Influenza [54], Retrovirus [55], Rabies virus [39]), this is not applicable here since culture medium was replaced with fresh medium during infection. As with other reports [56-59], more understanding on the virus replication mechanism is needed here to identify critical parameters.

### Production of ΔNS1 and wild type H1N1 Influenza virus in bioreactor

**Figure 6:**
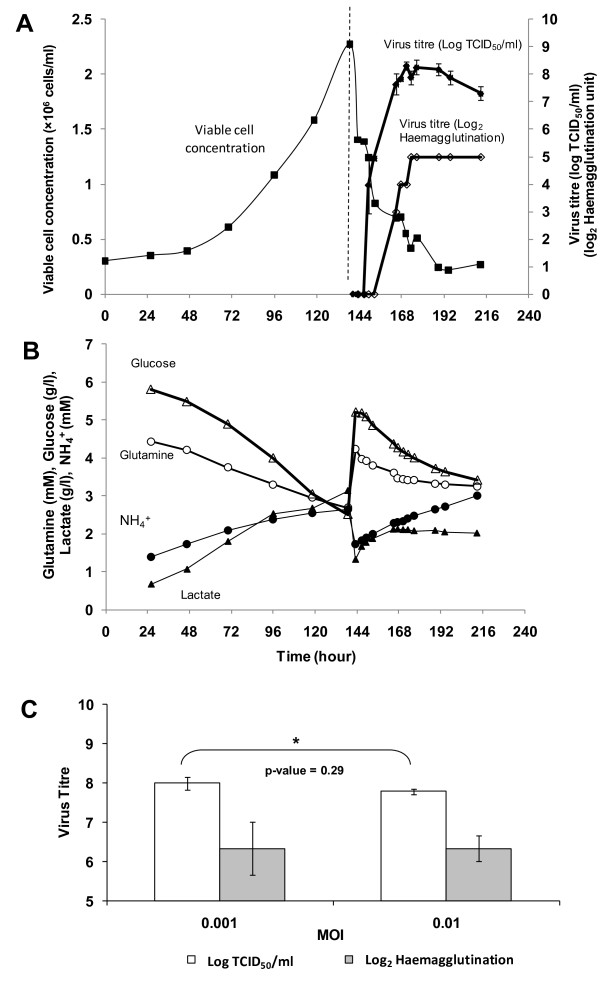
**Cell cultivation and ΔNS1 H1N1 Influenza virus amplification in 3 L stirred tank bioreactor using EX-CELL Vero SFM with 3 g/l Cytodex 1, MOI of 0.001 and trypsin concentration of 10 μg/ml**. (A) Cell growth profile and virus amplification of representative bioreactor run. Error bars indicate the standard error of triplicate measurements. (B) Metabolite profiles of glucose, lactate, glutamine and ammonium, before and after infection. (C) Comparison of max virus titres between infection at M.O.I 0.001 and 0.01 carried out in 3 L bioreactor scale. Log_2 _HAU is represented as filled bars and non-filled bar indicates virus titre in Log_10 _TCID_50_/ml. Error bars indicate the standard deviation of triplicate bioreactor runs.

The propagation of ΔNS1 H1N1 virus in bioreactor with EX-CELL Vero SFM containing Cytodex 1 is shown in Figure 6. Cells were infected with the virus when the cell concentration reached 2.3 × 10^6 ^cells/ml on day 6 with MOI of 0.001 and trypsin concentration of 10 μg/ml. The virus titres were monitored for 3 days. As shown in Figure 6A, upon infection viable cell concentration decreased rapidly which coincided with the increase in the virus titres. It reached the maximum of 8.3 Log_10 _TCID_50_/ml and 5 Log_2 _haemagglutination unit (HAU) 30 h after infection. The TCID_50 _titre then steadily decreased from 48 h post-infection onwards, suggesting that there was degradation of live virus particles in the bioreactor. As shown in Figure 6B, the virus production trends also correlate with the trends observed in the consumptions of glucose and glutamine and the productions of lactate and ammonia. Infection using a MOI of 0.01 showed comparable peak virus titres (Figure 6C), in agreement with our previous observations in small scale infections.

**Figure 7:**
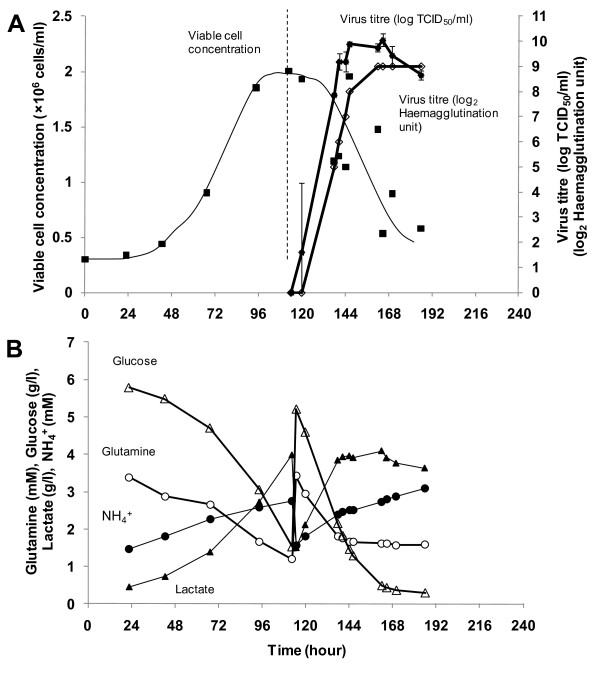
**Cell cultivation and wild type H1N1 Influenza virus amplification in 3 L stirred tank bioreactor using EX-CELL Vero SFM with 3 g/l Cytodex 1, MOI of 0.001 and trypsin concentration of 10 μg/ml**. (A) Cell growth profile and virus amplification of representative bioreactor run. Error bars indicate the standard error of triplicate measurements. (B) Metabolite profiles of glucose, lactate, glutamine and ammonium, before and after infection.

To compare ΔNS1 H1N1 virus production to the wild type Influenza virus, we performed bioreactor runs using the same media, identical bioreactor and infection parameters with the wild type Influenza A virus IVR-116, NIBSC code 06/108 corresponding to our model ΔNS1 H1N1 virus. The cell growth and the subsequent infection in EX-CELL Vero SFM are shown in Figure 7A. In metabolite profiles shown in Figure 7B, more glucose and glutamine were consumed and consequently more lactate was produced when compared to the profiles of ΔNS1 H1N1 in Figure 6B. The wild type virus titre of 10 Log_10 _TCID_50_/ml and 9 Log_2 _HAU was achieved in 24 hours.

**Figure 8:**
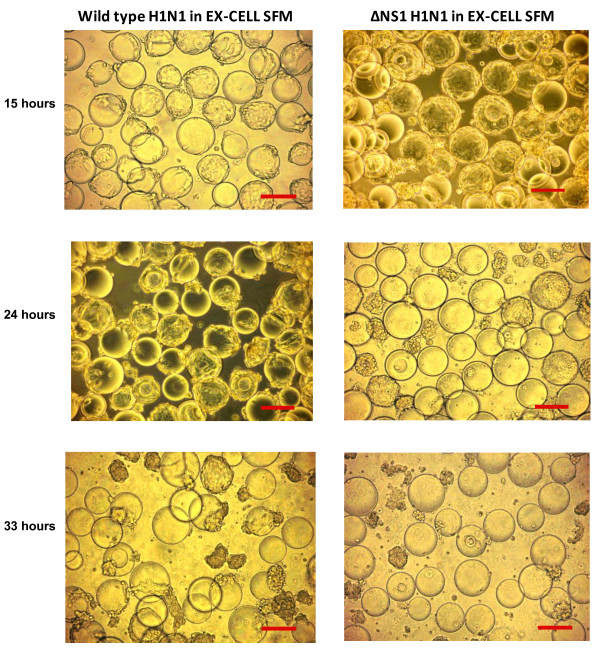
**Phase contrast images of cells cultured on Cytodex 1 microcarriers after infection with wild type and ΔNS1 H1N1 Influenza viruses in EX-CELL Vero SFM**. Scale bars indicate 200 μm.

The bioreactor processes for the production of ΔNS1 H1N1 and wild type H1N1 viruses were repeated at least twice to obtain average virus titres of 8.1 ± 0.30 Log_10 _TCID_50_/ml and 6.4 ± 0.40 Log_2 _HAU, and 9.6 ± 0.56 Log_10 _TCID_50_/ml and 8.5 ± 0.50 Log_2 _HAU respectively. The virus titres achieved in the wild type strain is 1.5 Log_10 _TCID_50_/ml higher and 2.1 Log_2 _HAU significantly higher (P < 0.05) than ΔNS1 H1N1 using EX-CELL Vero SFM. We speculate that this may be largely due to differences in the viruses, especially the NS1 deletion, since it was known that the NS1 protein in infected cells interacts with host cell gene expression and cellular protein regulation including interferon mediated antiviral responses [11,60,61]. Although the use of interferon deficient Vero cells allows us to circumvent the cellular antiviral responses [8,16], recent reports suggest that the Influenza A NS1 protein is also involved in anti-apoptotic signaling through Phosphatidylinositol-3-kinase (PI3K) pathway [62-64]. In agreement with literature, we observed that the viable cell concentration remained relatively unchanged during the first 24 h of the wild type virus infection and only started to decline when peak virus titre was reached (Figure 7A). In contrast, a more rapid decrease in viable cell concentrations upon infection with ΔNS1 strain was observed (Figure 6A). ΔNS1 H1N1 virus infected cells also showed more prominent cell death with earlier complete cell detachment from the microcarriers (Figure 8). We therefore believe that virus titre improvements may be possible via preventing apoptosis. A recent study by Seitz *et al*. [65] demonstrated the possibility of this approach with transient expressed NS1 gene in MDCK cells to enhance replication of an influenza virus lacking NS1.

Comparing our observed titres of the wild type H1N1 virus with literature, haemagglutination titre obtained in this study was 0.5 to 1 Log_2 _higher than other Vero bioreactor processes producing Influenza virus using serum-free or serum-containing media [33,35]. However this may not be significant considering haemagglutination activity varied between strains. In addition, these reports used avian erythrocytes for HA assay while we have used human erythrocytes. Our observed titres are also comparable to those reported in MDCK bioreactor processes producing Influenza viruses (2.4 to 3.3 Log_10 _HA/100 μl [26,27,35], 7.7 Log_10 _TCID_50_/ml [27], and 8.5 to 10 Log_10 _EID_50_/ml [28]).

### Comparison of ΔNS1 and wild type H1N1 Influenza virus production using EX-CELL Vero SFM and OptiPro SFM

As the ΔNS1 and wild type H1N1 Influenza virus titres were significantly different in EX-CELL Vero SFM, we wanted to find out whether this is also true in other SFM. In addition, we wanted to investigate whether the "cell density effect" can be observed when we compare EX-CELL Vero SFM with another serum-free medium that gives a lower maximum cell density. We thus performed the bioreactor virus production using OptiPro SFM for this comparison.

**Figure 9:**
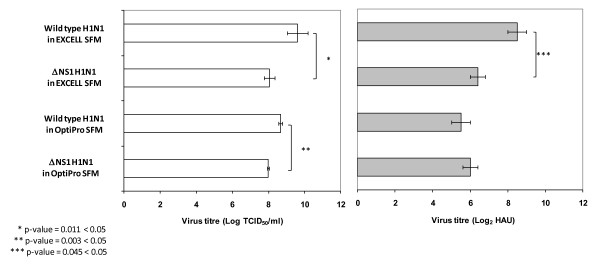
**Comparison of virus titres between ΔNS1 and the wild type H1N1 Influenza virus in EX-CELL Vero SFM and OptiPro SFM from 3 L stirred tank bioreactors**. Error bars indicate the standard deviation from at least two separate runs. Log_2_ HAU is represented as filled bars and non-filled bar indicates virus titre in Log_10_ TCID_50_/ml.

Using OptiPro SFM, the average virus titres of ΔNS1 H1N1 and wild type H1N1 viruses were 8.0 ± 0.05 Log_10 _TCID_50_/ml and 6.0 ± 0.41 Log_2 _HAU, and 8.7 ± 0.11 Log_10 _TCID_50_/ml and 5.5 ± 0.50 Log_2 _HAU respectively. Similar to the virus production process using EX-CELL Vero SFM, a higher TCID_50 _virus titre was obtained with the wild type virus compared to the ΔNS1 H1N1 virus, although the difference was less distinct than the results with EX-CELL Vero SFM and the haemagglutination titres were not significantly different (Figure 9). This confirms our previous observation in EX-CELL Vero SFM, that the inherent differences in the viruses, such as the deletion of the NS1 gene, may be the primary factor resulting in the different virus titres. We speculate that the difference is less distinct in OptiPro SFM due to the lower maximum cell density and peak virus titres, as discussed below.

Comparing the peak virus titres of the wild type virus in EX-CELL Vero SFM and OptiPro SFM, EX-CELL Vero SFM gave a 1 Log_10 _TCID_50_/ml higher titre, while that of the ΔNS1 H1N1 virus were similar (Figure 9). As cell concentration in EX-CELL Vero SFM was almost twice that of OptiPro SFM, this suggests that the higher cell density may be beneficial to increasing virus titre of the wild type H1N1 Influenza virus, contrary to the "cell density effect". On the other hand, the higher cell density achieved using EX-CELL Vero SFM did not contribute to a higher virus titre for the ΔNS1 H1N1 virus, validating our previous observation in TOI studies with the same virus (Figure 5). This suggests that the "cell density effect" observed for the ΔNS1 H1N1 virus may be due to differences between this virus and the wild type virus, and one obvious difference between these two viruses is the NS1 deletion. The absence of this gene may have allowed the virus to trigger apoptotic pathways in the Vero cells to limit virus yield, as discussed previously in this report.

To validate that the observed "cell density effect" is not due to changes in culture medium during virus production, we analyzed the metabolite profiles of these cultures. The analyses of glucose, glutamine and amino acids (data not shown) have not revealed any shortage during the virus production in the two SFM and two viruses tested. Ammonium concentrations were below 20 mM, while lactate concentrations were above 0.8 g/l prior to virus infection with both EX-CELL Vero SFM and OptiPro SFM. As it has been reported in MDCK cells that lactate concentration of 8 mM (or 0.7 g/l) at the time point for infection can reduce haemagglutination unit by a factor of two [66] and the addition of ammonium chloride to 20 mM was reported to block infection [67-69], the high lactate level we observed may have some influences to limit the maximum virus titres achieved with these medium. On the other hand, a higher lactate level was observed in the wild type virus production with 3.9 g/l generated 24 h post-infection. Hence it is unclear to what extent the virus production using Vero cells can be affected by lactate accumulation without further experiments.

## Conclusions

We have compared five commercially available SFM for the microcarrier based cultivation of Vero cells. In addition, we described for the first time the production of Influenza viruses using Vero cells in commercially available animal component-free, serum-free medium, and a potentially scalable stirred tank bioreactor process for the production of ΔNS1 H1N1 virus. Comparing the production of the ΔNS1 H1N1 virus to that of the corresponding wild-type strain, we showed that titres of ΔNS1 H1N1 virus were lower than that of the wild-type, and postulated that this may be a result of earlier Vero host cell death due to the NS1 deletion.

## Methods

### Preparation of cell line, virus strain, trypsin stock and microcarriers

Vero cells (ATCC CCL-81) from a master cell bank at passage 126 was thawed into DMEM (Invitrogen, Grand Island, NY) + 10% (v/v) fetal bovine serum (FBS) (Invitrogen) and passaged three times before stepwise adaptation to different serum-free media (SFM) to form working cell banks. The SFM used were OptiPro SFM (Invitrogen, Grand Island, NY, Cat. No. 12309-019), VP-SFM (Invitrogen, Cat. No. 11681-020), EX-CELL Vero SFM (SAFC Biosciences, Lenexa, KS, Cat. No. 14585), Provero-1 (Lonza, Belgium, Cat. No. BE-02-030Q) and HyQ SFM4MegaVir (HyClone, Logan, UT, Cat. No. SH-30522.01), supplemented according to manufacturers' instructions. Cells were subsequently thawed from these working cell banks and passaged in tissue culture flasks (T-flasks) in their respective media for 2 or more passages prior to use. Passage numbers of cells used for experiments were less than 150. Cells cultures were incubated in 37°C/5% CO_2 _humidified incubators (Sanyo, Japan).

Influenza A/New Caledonia/20/99(H1N1)-like virus, with NS1 deletion (ΔNS1) was provided by Avir Green Hills Biotechnology. The corresponding wild type Influenza A virus was obtained from NIBSC (Influenza IVR-116, NIBSC code 06/108). Working banks of the viruses were created by amplifying the virus using Vero cells cultivated in OptiPro SFM (Invitrogen) in T-flasks or Cell Factories (Nunc, Denmark). While the ΔNS1 H1N1 virus was already adapted to Vero cells, the wild type virus was propagated using Vero cells for 6 passages prior to the creation of the working bank. Virus titres of the ΔNS1 and wild type H1N1 working banks were 7.1 to 7.3 Log_10 _TCID50/ml and 4 to 5 Log_2 _HAU, and 9.0 Log_10 _TCID50/ml and 6.5 Log_2 _HAU respectively, quantified as described below.

Porcine trypsin used for Influenza virus activation (Sigma-Aldrich, St. Louis, MO, Cat. No. T5266, 1500 BAEE unit/mg) was dissolved in deionized water and sterile filtered to make a 5 mg/ml stock solution. This stock solution was aliquoted and stored in -20°C freezer. Trypsin aliquots were thawed once for experiments.

Cytodex 1 microcarriers (GE Healthcare, Sweden, Cat. No. 17-0448-01) were hydrated and sterilized in a glass bottle pre-coated with Sigmacote^® ^(Sigma-Aldrich, Cat. No. SL2) according to manufacturers' instructions.

### Quantification of virus titres and monitoring of cell cultivation process

Virus titres were quantified using the haemagglutination assay [70] and tissue culture infectious dose (TCID_50_) assay [53]. For the haemagglutination assay, 4% human erythrocytes (Siemens Healthcare Diagnostics, Germany) were diluted 8-fold in Dulbecco's phosphate buffer solution (PBS, Invitrogen, Cat. No. 14190-250) to obtain a 0.5% cell suspension. 50 μl of this 0.5% cell suspension was then added to an equal volume of virus and control samples in 2-fold serial dilutions. Haemagglutination unit (HAU) of a virus sample was read as the highest dilution in which haemagglutination was observed.

TCID_50 _assay was performed by adding 50 μl of 10-fold serially diluted virus samples to Vero cells cultivated on 96 well plates using OptiPro SFM (Invitrogen) supplemented with 5 μg/ml porcine trypsin (Sigma-Aldrich). The assay was carried out in triplicate sets, each consisting of 6 wells per diluted virus sample. TCID_50 _titres were calculated according to the formula of Reed and Muench [71]. As the dose for attenuated influenza virus vaccine in clinical studies is measured based on TCID_50 _assay [72,73], the comparisons of virus titres in our report were predominantly based on TCID_50 _instead of haemagglutination assay.

Cell cultivation process was monitored by measuring total cell density, viable cell density and key metabolite concentrations. Total cell density was determined with crystal violet nuclei staining. Briefly, a 500 μl aliquot of cell culture sample was treated with an equal volume of 0.01% (w/v) crystal violet (Sigma-Aldrich) in 0.1M citric acid solution and incubated for 30-45 minutes at 37°C. The released nuclei from the cells were then counted using a hemacytometer. To determine viable cell density, we first observed that cells attached to the microcarriers were mostly viable with cell viability greater than 90% regardless of the stage of cell cultivation (data not shown). Based on this observation, Trypan blue cell exclusion method was used to obtain the density of non-viable cells that were not attached to microcarriers. Viable cell density was then estimated by deducting this from the total cell density measured with crystal violet nuclei staining.

Concentrations of key metabolites (glutamine, glucose, lactate and ammonium) in cell culture supernatants were analyzed using BioProfile 100 Plus (Nova Biomedical, Waltham, MA) as per manufacturer's instructions. Virus-containing samples were deactivated by heating in a 60°C water bath for 30 minutes prior to analysis.

### Cell cultivation and virus infection in spinner flasks

250 ml spinner flasks (Bellco, Vineland, NJ) were coated with Sigmacote (Sigma-Aldrich) according to manufacturer's instructions. Vero cells were seeded into the spinner flasks at 6 × 10^5 ^cells/ml in 125 ml medium containing 6 mg/ml of hydrated Cytodex 1 microcarriers. Mixing in the spinner flasks was performed using a magnetic stirrer platform (Cellgro Type 45600, Thermolyne, Dubuque, IA) which rotates the magnetic microcarrier paddle impeller (Bellco, Cat No. 1965-30100) inside the flasks. The spinner flasks were stirred at 40 rpm inside a 37°C/5% CO_2 _humidified incubator. Surface aeration was allowed by loosening the cap on one arm of the spinner flask. After 24 h, the stirring speed was increased to 60 rpm, and the culture volume was increased to 250 ml by addition of fresh medium. This results in cell and microcarrier concentrations of 3 × 10^5 ^cells/ml and 3 mg/ml respectively.

Prior to influenza virus infection, approximate 80% of the culture medium was exchanged. Influenza virus at predetermined MOI and trypsin were added to the spinner flask together with fresh medium. Stirring speed was then maintained at 60 rpm.

To determine the conditions for infection, cells were first cultivated on microcarriers in the spinner flasks as described above. At various stages of cultivation, the cells were transferred to suspension 6-well plates (Greiner Bio-One, Cat no. 657102) with 5 ml culture volume per well. The well was incubated in 37^°^C/5% CO_2 _humidified incubators (Sanyo, Japan) on orbital shaker agitated at speed of 100 rpm. Different virus infection conditions, namely time of infection, trypsin concentration, and MOI, were tested in this format.

### Cell cultivation and virus infection in 3 L bioreactor

3 L bioreactor (Applikon, Netherlands) was coated with Sigmacote^® ^(Sigma-Aldrich) and sterilized according to manufacturer's instructions. The bioreactor settings for experiments were as follows: culture temperature was set at 37°C; maximum flow rate for O_2 _and CO_2 _were set at 10 ml/min; dissolved oxygen (DO) was set at 40% saturated air concentration; initial stirring speed was 60 rpm; initial pH were 7.1 and 7.3 for EX-CELL Vero SFM and OptiPro SFM respectively. Vero cells were seeded at 6 × 10^5 ^cells/ml in 750 ml medium with a microcarrier density of 6 mg/ml. At 6 h post-seeding, the pH and stirring speed were changed to 7.2 and 100 rpm respectively, as most cells were attached to the microcarriers. At 24 h post-seeding, the culture volume was increased to 1.5 L by adding fresh medium, to result in cell and microcarrier concentrations of 3 × 10^5 ^cells/ml and 3 mg/ml respectively.

Influenza virus infection in bioreactor is similar to that in spinner flasks. 80% of the culture medium was first exchanged with fresh medium. Influenza virus at predetermined MOI and trypsin were then added to the bioreactor together with fresh medium. For infection in OptiPro SFM, 5 μg/ml trypsin was used, and the cells were infected at MOI 0.001 and 0.01 at cell concentrations of 1.2-1.4 × 10^6 ^cells/ml. The bioreactor settings remained unchanged.

### Statistical analysis

Statistical analysis was carried out using two-tailed student's t-tests assuming equal variance with replications. P values less than 0.05 were considered significant.

## Authors' contributions

AC participated in the design of the study, performed the statistical analysis and drafted the manuscript. SLP carried out the virus quantifications and cell culture studies, and participated in equipment set up and material preparations. CD participated in the design of the study, in equipment set up and in parallel experiment verification (not presented). ER participated in the design of the study, in equipment set up and material preparations. MLY participated in the virus quantifications and cell culture studies, and in equipment set up and material preparations. SKN conceived the study, participated in its design and coordination, and drafted the manuscript. All authors read and approved this final manuscript.
